# A Classifier for Improving Early Lung Cancer Diagnosis Incorporating Artificial Intelligence and Liquid Biopsy

**DOI:** 10.3389/fonc.2022.853801

**Published:** 2022-03-02

**Authors:** Maosong Ye, Lin Tong, Xiaoxuan Zheng, Hui Wang, Haining Zhou, Xiaoli Zhu, Chengzhi Zhou, Peige Zhao, Yan Wang, Qi Wang, Li Bai, Zhigang Cai, Feng-Ming (Spring) Kong, Yuehong Wang, Yafei Li, Mingxiang Feng, Xin Ye, Dawei Yang, Zilong Liu, Quncheng Zhang, Ziqi Wang, Shuhua Han, Lihong Sun, Ningning Zhao, Zubin Yu, Juncheng Zhang, Xiaoju Zhang, Ruth L. Katz, Jiayuan Sun, Chunxue Bai

**Affiliations:** ^1^ Department of Pulmonary and Critical Care Medicine, Zhongshan Hospital, Fudan University, Shanghai, China; ^2^ Shanghai Respiratory Research Institute, Shanghai, China; ^3^ Department of Respiratory Endoscopy, Shanghai Chest Hospital, Shanghai Jiao Tong University, Shanghai, China; ^4^ Department of Respiratory and Critical Care Medicine, Shanghai Chest Hospital, Shanghai Jiao Tong University, Shanghai, China; ^5^ Xinxiang Medical University, Xinxiang, China; ^6^ Department of Respiratory and Critical Care Medicine, Henan Provincial People’s Hospital, People’s Hospital of Zhengzhou University, Zhengzhou, China; ^7^ Department of Thoracic Surgery, Respiratory Center of Suining Central Hospital, Suining, China; ^8^ Department of Pulmonary and Critical Care Medicine, Zhongda Hospital, Southeast University, Nanjing, China; ^9^ State Key Laboratory of Respiratory Disease, National Clinical Research Center of Respiratory Disease, Guangzhou Institute of Respiratory Health, First Affiliated Hospital of Guangzhou Medical University, Guangzhou, China; ^10^ Department of Respiratory and Critical Care Medicine, Affiliated Hospital of Qingdao University, Qingdao, China; ^11^ Department of Respiratory and Critical Care Medicine, Liaocheng People’s Hospital, Liaocheng, China; ^12^ Department of Respiratory Medicine, The Second Affiliated Hospital of Dalian Medical University, Dalian, China; ^13^ Department of Respiratory Disease, Xinqiao Hospital, Army Medical University, Chongqing, China; ^14^ The First Department of Pulmonary and Critical Care Medicine, The Second Hospital of Hebei Medical University, Shijiazhuang, China; ^15^ Clinical Oncology Center, The University of Hong Kong-Shenzhen Hospital, Shenzhen, China; ^16^ Department of Respiratory Medicine, The First Affiliated Hospital, College of Medicine, Zhejiang University, Hangzhou, China; ^17^ Department of Epidemiology, College of Preventive Medicine, Army Medical University, Chongqing, China; ^18^ Division of Thoracic Surgery, Zhongshan Hospital, Fudan University, Shanghai, China; ^19^ Joint Research Center of Liquid Biopsy in Guangdong, Hong Kong, and Macao, Zhuhai, China; ^20^ Zhuhai Sanmed Biotech Ltd., Zhuhai, China; ^21^ Department of Thoracic Surgery, Xinqiao Hospital, Army Medical University, Chongqing, China; ^22^ Chaim Sheba Hospital, Tel Aviv University, Ramat Gan, Israel

**Keywords:** lung cancer, artificial intelligence, liquid biopsy, prediction model, early diagnosis

## Abstract

Lung cancer is the leading cause of cancer-related deaths worldwide and in China. Screening for lung cancer by low dose computed tomography (LDCT) can reduce mortality but has resulted in a dramatic rise in the incidence of indeterminate pulmonary nodules, which presents a major diagnostic challenge for clinicians regarding their underlying pathology and can lead to overdiagnosis. To address the significant gap in evaluating pulmonary nodules, we conducted a prospective study to develop a prediction model for individuals at intermediate to high risk of developing lung cancer. Univariate and multivariate logistic analyses were applied to the training cohort (n = 560) to develop an early lung cancer prediction model. The results indicated that a model integrating clinical characteristics (age and smoking history), radiological characteristics of pulmonary nodules (nodule diameter, nodule count, upper lobe location, malignant sign at the nodule edge, subsolid status), artificial intelligence analysis of LDCT data, and liquid biopsy achieved the best diagnostic performance in the training cohort (sensitivity 89.53%, specificity 81.31%, area under the curve [AUC] = 0.880). In the independent validation cohort (n = 168), this model had an AUC of 0.895, which was greater than that of the Mayo Clinic Model (AUC = 0.772) and Veterans’ Affairs Model (AUC = 0.740). These results were significantly better for predicting the presence of cancer than radiological features and artificial intelligence risk scores alone. Applying this classifier prospectively may lead to improved early lung cancer diagnosis and early treatment for patients with malignant nodules while sparing patients with benign entities from unnecessary and potentially harmful surgery.

**Clinical Trial Registration Number:**

ChiCTR1900026233, URL: http://www.chictr.org.cn/showproj.aspx?proj=43370.

## Introduction

Approximately 22% of the newly diagnosed cancer cases worldwide and 27% of cancer-related deaths occur in China (1). In 2018, the 5-year survival rate for lung cancer in China was 19.7% (2). Based on the results of the National Lung Screening Trial (NLST) (3, 4), low-dose computed tomography (LDCT) is the recommended test for lung cancer screening, but the high false-positive rate has diminished the benefits of the test; indeed, in a previous study, only 3.6% of the participants who had pulmonary nodules were confirmed to have lung cancer (3). Therefore, clinicians use diagnostic decision tools to stratify the malignancy risk of patients with positive LDCT results (5). The Mayo Clinic Model has been extensively validated worldwide and includes factors such as age, smoking history, extra-thoracic cancer history, spiculation, nodule diameter, and upper lobe location (6). However, because of the variation in ethnicity and environment, some risk factors might have different impacts on the Chinese population. For example, the diagnostic significance of the malignant risk factor “upper lobe location” is weakened owing to the high prevalence of tuberculosis (7).

New technologies have resulted in the emergence of several tools for early cancer diagnosis. Artificial intelligence (AI) approaches combined with deep learning technology have been adopted for image analysis in clinical settings. The use of AI can help clinicians reduce the risk of human errors caused by classifying a large number of medical images (8), which may lead to improved diagnostic efficacy of LDCT for lung cancer (9). Several studies have demonstrated that the application of deep learning technology may improve the performance of lung cancer diagnosis by the precise recognition of specific malignant features from LDCT images (10, 11). In general, AI can analyze the whole pulmonary nodule, looking for features characteristic of invasion, as opposed to histopathological evaluation of a small biopsy taken from an intermediate- or high-risk pulmonary nodule, which may not be representative (8, 11, 12). In addition, testing for early lung cancer *via* liquid biopsy using novel, sensitive, and specific biomarkers to examine cancer-related proteins or abnormal DNA (13, 14). Liquid biopsy for early lung cancer detection has been extensively investigated with various biomarkers and platforms. Indeed, previous studies (15–17) demonstrated that a fluorescent *in situ* hybridization (FISH) liquid biopsy approach to detect cells with cytogenetic abnormalities may be used to rule out lung cancer in individuals with intermediate pulmonary nodules (18, 19).

Guidelines for the early diagnosis of lung cancer in China recommend that prediction models be established based on data retrieved from Chinese populations (20), based on a broad range of preliminary information and evidence (21, 22). We hypothesized that the integration of clinical and radiological characteristics, together with AI interpretation of LDCT images and liquid biopsy testing for cells with cytogenetic abnormalities *via* a 4-color FISH array, might improve the ability to diagnose early lung cancer in individuals with intermediate and high-risk pulmonary nodules on LDCT. To this end, we conducted a prospective multicenter study in China to establish an effective early lung cancer prediction model to improve the diagnosis of pulmonary nodules with an intermediate and high risk of lung cancer detected by LDCT.

## Material and Methods

### Study Population

**Figure 1:**
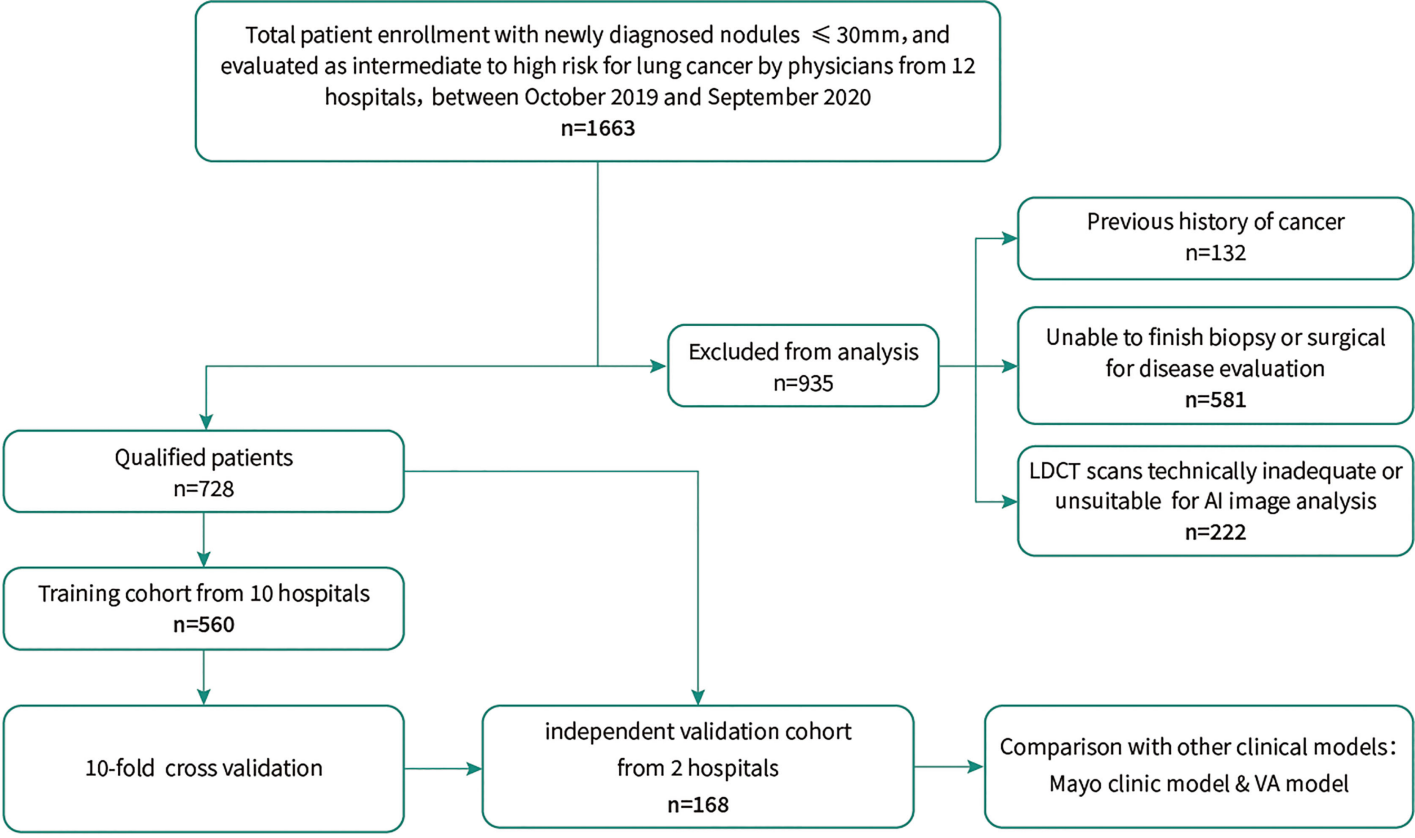
Schematic Diagram of the Study Design.

The study was approved by the Institutional Review Board of Zhongshan Hospital of Fudan University. A total of 1,663 individuals were recruited to the study from consecutive outpatients of 12 tertiary hospitals across mainland China. Pulmonary nodules detected by LDCT were identified as intermediate and high-risk for lung cancer by physicians in the usual care routine. Intermediate risk was defined as individuals requiring follow up to rule out malignancy, while high-risk was defined as individuals with a clinical suspicion of lung cancer. The flow chart in **Figure 1** describes the criteria for patient recruitment in this study. Written informed consent was obtained from all participants.

Eligible patients recruited from ten hospitals between September 2019 and September 2020 were enrolled in the training set to establish an early lung cancer prediction model. Subsequently, an independent validation set composed of participants evaluated between March 2020 and October 2020 from the remaining two hospitals was used to test the diagnostic performance of the comprehensive lung cancer risk prediction model. The final selection of the individuals comprising the training set (n = 560) and independent validation set (n = 168) was based on the exclusion criteria shown in **Figure 1**.

### Data Collection

All participants completed a demographic survey to obtain clinical information. LDCT images in the 6 months prior to enrollment of individuals were obtained for AI analysis. Following AI of LDCT scans and liquid biopsy, patients with intermediate and high-risk pulmonary nodules who met the inclusion criteria were subjected to fiberoptic bronchoscopy, fine needle biopsy, and/or surgical resection of their nodules for pathological examination. The World Health Organization classification for lung tumors was used to classify lung masses, and staging was based on the 8th edition of the TNM Classification for Lung Cancer of the International Cancer Control and the American Joint Committee on Cancer staging system.

### AI Analysis Tool Development

**Figure 2:**
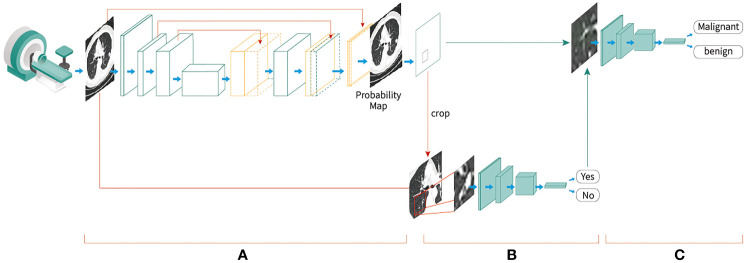
End-to-end deep convolutional neural network-based Artificial Intelligence low-dose computed tomography analysis toll development procedures, **(A)** A three-dimensional (3D) U-net-based convolutional neural network was used for the segmentation of lung nodules to identify suspicious nodules; **(B)** the 3D patches of the suspicious nodules were cropped and forwarded to a false-positive reduction network to discriminate the true clinically positive nodules from the false-positive nodules; **(C)** the patches that were labeled as positive were forwarded to a convolutional neural network-based classifier to determine whether the nodule was malignant or benign.

An automated diagnostic platform comprising a deep-learning-based AI algorithm with a three-stage end-to-end deep conventional neural network (DCNNs) was developed to analyze the LDCT images of the patients. First, a 3D U-net-based DCNN was used for the patch segmentation of lung nodules to identify suspicious nodules. The LDCT images with labels were cropped in a sliding window style and feed into a 3-layer 3D U-Net segmentation model for training. Then the predicted segmentation patches were combined to generate final segmentation results. Next, the 3D patches of the suspicious nodules were forwarded to a false positive reduction network (FPRN) to discriminate the true clinically positive nodules from the false positive nodules. Then, the patches that were labeled positive were forwarded to a CNN-based classifier to determine whether the nodule was malignant or benign. This 3D U-net segmentation network was initially trained with the publicly available The Lung Image Database Consortium and Image Database Resource Initiative (LIDC-IDRI) dataset and then further trained on a dataset of about approximately 20,000 samples from hospitals in the U.S. and China with histopathological results. Through further evaluation by experienced radiologists, the patches identified by the U-net in the first stage were segmented by manually marking the true clinically positive nodules and false positive nodules. The FPRN and malignant/benign (M/B) classifier were then trained at the patch level according to the true malignancy status confirmed by pathology results (**Figure 2**). All networks were trained with Python 3.6 and Tensorflow 1.10 on a NVIDIA DGX station. The LDCT data of the 728 participants were saved in DICOM format and uploaded to the AI lung nodule analysis platform for analysis. After the images were analyzed, the AI model provided a risk score for developing lung cancer (ranging from 0 to 100%) and a diagnosis statement for each participant.

### Liquid Biopsy

**Figure 3:**
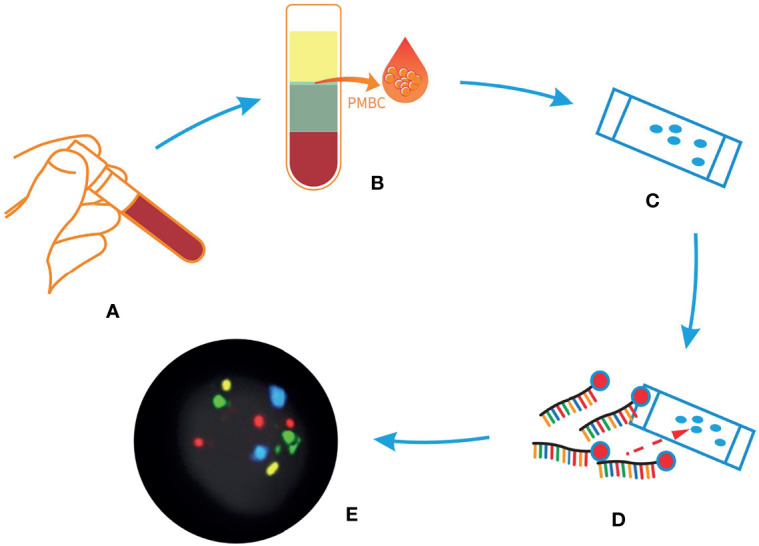
Sample process procedures of liquid biopsy *via* 4-color fluorescent *in situ* hybridization (FISH) assay. **(A)** Peripheral blood from patients with indeterminate or high-risk nodules. **(B)** The peripheral blood mononuclear cells layer was isolated after configuration. **(C)** The peripheral blood mononuclear cells were applied to a glass slide. **(D)** Hybridization with 4-color FISH probes. **(E)** The result of the assay, scanned with a Duet microscope system.

To detect genetically circulating abnormal cells, we used a peripheral blood 4-color FISH assay developed to generate data for this study (23). This multiplex interphase FISH assay is composed of four DNA probes that are universally deleted in non-small cell lung cancer (NSCLC) and have been implicated in the pathogenesis of NSCLC (14, 23). This assay has previously shown a high degree of accuracy in detecting cells containing chromosomal abnormalities at *10q22.3* and *3p22.1* and in the internal control genes *CEP 10* and *3q29* (14) in several studies involving the detection of early lung cancer (24). Abnormal cells that were discovered by the 4-color FISH assay were identified as intact cells with a nucleus larger than a lymphocyte nucleus and polysomy of at least two probes per nucleus. The FISH assay was performed according to the manufacturer’s instructions as previously described (**Figure 3**) (25).

### Statistical Analysis

Descriptive analyses of the variables are expressed as means, ranges, or numbers, expressed as percentages (%). Statistical analysis was performed using Python version 3.8.5 (Python Software Foundation, USA) and MedCalc version 19.0.4 (MedCalc Software Ltd., Ostend, Belgium). All tests were 2-sided, and statistical significance was set at p <0.05.

Receiver operating curves (ROCs) were used to determine the individual performance of AI and liquid biopsy using the 4-color FISH assay. Univariate logistic regression analyses were used to determine the individual factors associated with early lung cancer in the training cohort. Variables with p <0.05 in the univariate analysis were included in a multivariate logistic regression analysis to examine the independent predictive factors for inclusion in the early lung cancer diagnostic models with different sets of predictors. Cohen’s kappa (κ) statistic was used to measure the reliability of the individual predictors. The mean sensitivity, specificity, and area under the curve (AUC) from the 10-fold cross validation were used to determine the diagnostic power of multiple early lung cancer prediction models. Sensitivity and specificity were used to evaluate the ability of the best-performing model to classify malignancy in an independent validation cohort. AUCs were also applied to display the classification performance of the individual validation set in Model 4, the Mayo Clinic Model, and the Veteran Affairs (VA) model.

## Results

### Patient Characteristics

**Table 1** describes the clinical characteristics of the training and independent validation cohorts according to whether the underlying pathology was benign or malignant.

**Table 1:** Clinical characteristics of the study participants.

Variables		Benign Nodule		Malignant Nodule	
		Training Cohort	Validation Cohort	Training Cohort	Validation Cohort
		n = 135	n = 63	n = 425	n = 105
Age, y, mean, range		55 (18–81)	57 (30–82)	60 (25–82)	57 (25–81)
Sex, no. of participants (%)					
Male		76 (56%)	37 (59%)	204 (48%)	46 (44%)
Female		59 (44%)	26 (41%)	221 (52%)	59 (56%)
Smoking history, no. of participants (%)					
Current or past smoker^		25 (19%)	17 (27%)	251 (59%)	73 (70%)
Nonsmoker		110 (81%)	46 (73%)	174 (41%)	32 (30%)
Family history, no. of participants (%)					
Yes		8 (6%)	24 (38%)	42 (10%)	39 (37%)
No		127 (94%)	39 (62%)	383 (90%)	66 (63%)
Diameter of the nodule, millimeter, mean, range		12 (1–29)	9 (2–23)	17 (1–30)	14 (4–30)
Nodule count, no. of participants (%)					
Single		80 (59%)	19 (30%)	335 (79%)	33 (31%)
Multiple		55 (41%)	44 (70%)	90 (21%)	72 (69%)
Type of nodule, no. of participants (%)					
Solid		97 (72%)	27 (43%)	207 (49%)	20 (19%)
Subsolid		38 (28%)	36 (57%)	218 (51%)	85 (81%)
Nodule location, no. of participants (%)					
Upper lobe		61 (45%)	24 (38%)	251 (59%)	68 (65%)
Non-upper lobe		74 (55%)	39 (62%)	174 (41%)	37 (35%)
Nodule edge, no. of participants (%)					
Entirely smooth		85 (63%)	35 (56%)	144 (34%)	16 (15%)
Malignant signs*		50 (37%)	28 (44%)	281 (66%)	89 (85%)
Malignant subtypes					
Adenocarcinoma				361 (85%)	97 (92%)
Squamous cell carcinoma				23 (5%)	3 (3%)
Others				41 (10%)	5 (5%)
Cancer stage					
IA1				103 (24%)	45 (43%)
IA2				176 (42%)	45 (43%)
IA3				146 (34%)	15 (14%)

^Current and past smokers were identified as 20 pack-years and a quit time of <15 years, respectively.

*Signs of malignancy indicate nodules with one or more of the following: lobulation, spiculation, vacuole sign, pleural indentation, vessel convergence sign, or other radiological signs of malignancy.

### Diagnostic Performance of the AI Risk Score and Liquid Biopsy

We evaluated the diagnostic ability of the AI risk score and liquid biopsy results to discriminate between benign and malignant nodules. According to the Youden index, the AI risk score had the best performance when the threshold value was set to >71%. This threshold was associated with a sensitivity of 73.77% (95% confidence interval [CI]: 69.81–77.47%) and a specificity of 65.15% (95% CI: 58.07–71.77%) in the overall cohort.

**Figure 4:**
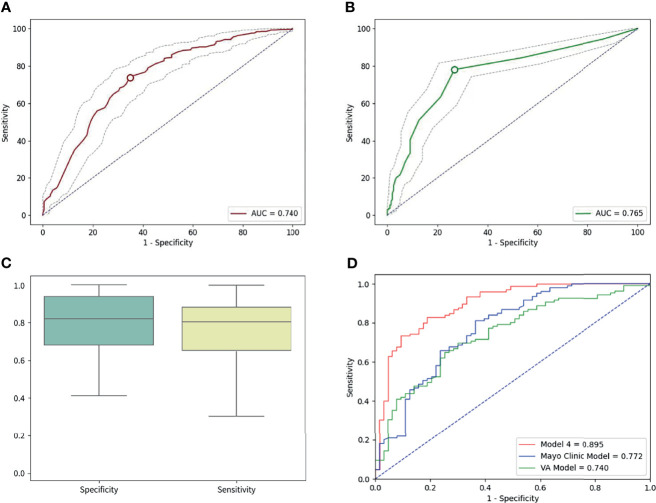
**(A)** The area under the curve (AUC) of AI was 0.740 in the overall cohort. **(B)** The AUC of liquid biopsy was 0.765 on the overall cohort. **(C)** The sensitivity was 82.8%, and the specificity was 80.95 in the independent validation cohort for the best performing model (model 4). **(D)** In the validation cohort, the areas under the curve were 0.895, 0.772, and 0.740 for model 4, the Mayo Clinic Model, and the VA model, respectively.

Similarly, when the cutoff value for the number of abnormal cells was set to ≥3, the sensitivity and specificity were 78.11% (95% CI: 74.35–81.56%) and 73.23% (95% CI: 66.49–79.26%), respectively. Based on the ROC curves of both tools, the AUC was 0.740 (95% CI: 0.698–0.782) for the AI risk score and 0.765 (95% CI: 0.727–0.803) for liquid biopsy in the overall cohort (**Figure 4**). Weak internal validity between the AI risk score and liquid biopsy data (κ = 0.16, 95% CI: 0.072–0.247) was observed, indicating the good complementary value of the two tools in early lung cancer diagnosis.

### Relationship Between Individual Predictors and Lung Cancer

Next, individual radiological and clinical predictive factors were evaluated in a univariate logistic regression analysis using data from 560 patients in the training cohort. It was demonstrated that nodule diameter (p <0.001), nodule count (p <0.001), subsolid status (p <0.001), upper lobe location (p = 0.005), and malignant features, namely, lobulation, spiculation, vacuole sign, pleural indentation, and vessel convergence sign or other radiological malignant signs at the nodule edge (p <0.001), were independent radiological predictors of malignancy. Age (p <0.001), current smokers with 20 pack-years, or past smokers with quit time <15 years (p <0.001) were clinical characteristics that correlated with lung cancer. Both the risk score predicted by AI LDCT image analysis (p <0.001) and quantitation of abnormal cells identified by liquid biopsy (p <0.001) were strongly associated with malignancy (**Table 2**).

**Table 2:** Univariate analyses of predictors of malignancy.

Variable	Odds Ratio(95% CI)	P-value
Age*	1.041 (1.024–1.059)	<0.001
Sex	0.717 (0.485–1.058)	0.094
Current or past smoking*	6.347 (3.946–10.210)	<0.001
Family history	1.741 (0.796–3.806)	0.165
Nodule diameter*	1.106 (1.073–1.140)	<0.001
Nodule count*	0.786 (0.703–0.879)	<0.001
Subsolid status*	2.713 (1.780–4.133)	<0.001
Upper lobe*	1.750 (1.85–2.585)	0.005
Malignant signs at the nodule edge*	3.247 (2.159–4.882)	<0.001
AI risk score*	36.891 (15.745–86.441)	<0.001
Liquid biopsy result*	1.379 (1.260–1.511)	<0.001

*Indicates significantly associated with lung cancer.

### Multivariate Logistic Regression Analysis to Build Early Lung Cancer Prediction Models

**Figure 5:**
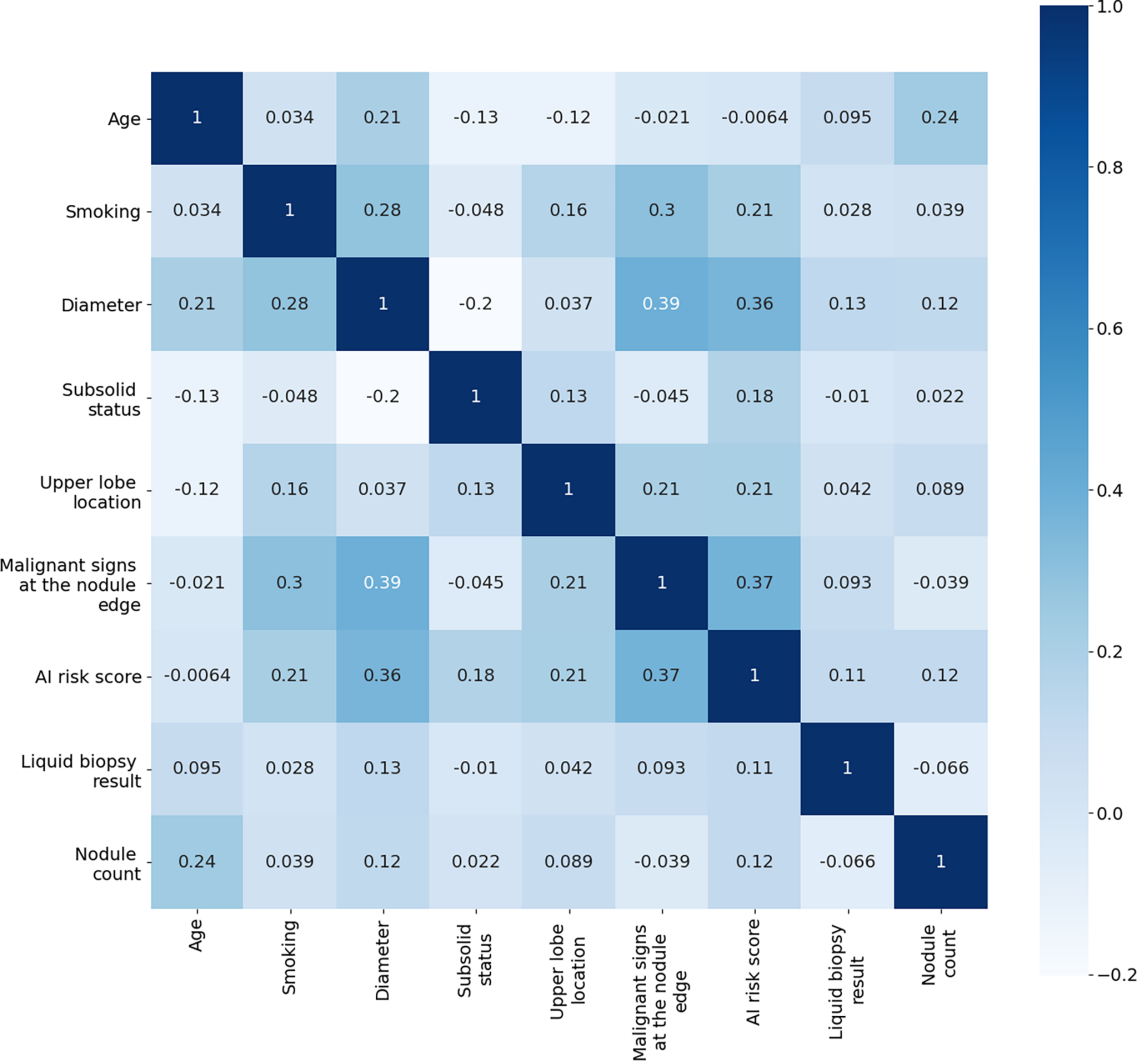
Correlation Heat Map of Individual Predictors in the Training Cohort.

Before building the early lung cancer prediction models, we applied correlation analyses to test the internal validation of the individual early lung cancer risk predictors. The correlation heat maps showed that the correlations between age, smoking, AI risk factors, liquid biopsy results, and radiological predictors that were significantly associated with malignancy in the univariate analysis were very weak (**Figure 5**), revealing that there was no multicollinearity association between each predictor.

Using multivariate logistic regression analysis based on the malignancy predictors identified using the univariate statistical method, we first built four models, each with a different set of predictors (**Table 3**). Next, we calculated the diagnostic powers of the four models using 10-fold cross validation. The lowest diagnostic performance was found in model 1, which comprised only radiological characteristics (diameter, nodule count, subsolid status, upper lobe location, and malignant signs at the nodule edge), with sensitivity, specificity, and AUC of 89.01% (95% CI: 82–96.03%), 62.52% (95% CI: 50.33–74.70%), and 0.769 (95% CI: 0.719–0.820), respectively. In model 2, when predictors were also consistent with radiological characteristics, with the addition of the AI risk score, there was a slight increase in the AUC to 0.791 (95% CI: 0.737–0.845), with a sensitivity of 89.18% (95% CI: 81.30–97.09%) and a specificity of 65.96% (95% CI: 53.13–78.80%). For model 3, we attempted to integrate clinical characteristics (age and smoking), radiological characteristics, and the quantitation of abnormal cells identified by the 4-color FISH test to determine the power of the risk prediction model without AI. The AUCs of model 3 achieved 0.872 (95% CI: 0.846–0.900), with 86.29% (95% CI: 77.32–95.25%) sensitivity and 83.25% (95% CI: 76.70–89.80%) specificity. The best diagnostic performance appeared to be model 4, which combined clinical and radiological characteristics, the AI risk score, and liquid biopsy results, with 89.53% (95% CI: 81.79–97.26%) sensitivity, 81.31% (95% CI: 76.43–86.18%) specificity, and an AUC of 0.880 (95% CI: 0.852–0.910), respectively (**Table 3**).

**Table 3:** Ten-fold cross validation result of classifiers with different predictors.

	Predictors	Sensitivity (mean, 95% CI)	Specificity (mean, 95% CI)	AUC (mean, 95% CI)
Model 1	Diameter + nodule count + subsolid status + upper lobe location + malignant signs at the nodule edge	89.01% (82.00–96.03%)	62.52% (50.33–74.70%)	0.769 (0.719–0.820)
Model 2	Diameter + nodule count + subsolid status + upper lobe location + malignant signs at the nodule edge + AI risk score	89.18% (81.30–97.09%)	65.96% (53.13–78.80%)	0.791 (0.737–0.845)
Model 3	Age + smoking + diameter + nodule count + subsolid status + upper lobe location + malignant signs at the nodule edge + liquid biopsy result	86.29% (77.32–95.25%)	83.25% (76.70–89.80%)	0.872 (0.846–0.900)
Model 4	Age + smoking + diameter + nodule count + subsolid status + upper lobe location + malignant signs at the nodule edge + AI risk score + liquid biopsy result	89.53% (81.79–97.26%)	81.31% (76.43–86.18%)	0.880 (0.852–0.910)

CI, confidence interval; AUC, area under the curve.

### Performance of the Best Model in Independent Validation Cohort & Comparison With Other Clinical Models

Based on the perimeters that we developed from the training cohort, we tested the power of the best early lung cancer prediction model that combined clinical characteristics (age and smoking), radiological characteristics (diameter, nodule count, subsolid status, upper lobe location, and malignant signs at the nodule edge), AI risk score, and liquid biopsy results of the 4-color FISH assay in the independent validation cohort (n = 168) (**Table 1**). This model reached 82.86% (95% CI: 74.27–89.51%) sensitivity and 80.95% (95% CI: 69.09–89.75%) specificity for classifying malignant and benign nodules. ROC calculations on model 4, the Mayo Clinic Model, and the VA model were utilized. The AUCs of model 4 were 0.895 (95% CI: 0.844–0.946) in the same cohort compared to 0.772 for the Mayo Clinic Model (95% CI: 0.696–0.848) and 0.740 (95% CI: 0.663–0.817) for the VA model (**Figure 4**).

## Discussion

In this prospective Chinese cohort study, clinical and radiological characteristics, together with the AI risk score of LDCT image analysis and quantitation of abnormal cells detected *via* a 4 color FISH-based liquid biopsy assay, were used to build an early lung cancer prediction model to diagnose malignant pulmonary nodules in individuals evaluated as having an intermediate and high risk of lung cancer from outpatient clinics at 12 tertiary hospitals across China with newly diagnosed pulmonary nodules. Our study was a diagnostic study and not a screening study as the study population did not comprise a typical screening population with the set criteria according to the NLST. Instead, we focused on detecting lung cancer in individuals with intermediate and high-risk pulmonary nodules as confirmed by pathological examination following subsequent surgical resection. The training set was comprised of data from 560 patients and was used to establish the model. Subsequently, the efficacy of the model was tested in a validation study using data from a different set of 168 participants. We only included patients with pulmonary nodules ≤30 mm, which means that individuals with malignant pulmonary nodules were all diagnosed with stage IA (T1N0M0) lung cancer according to the TNM classification.

To the best of our knowledge, this may be one of the first studies to integrate AI for LDCT image analysis and liquid biopsy to build a prediction model to diagnose malignant pulmonary nodules in individuals with intermediate and high risks of lung cancer in a prospective cohort. We observed an improvement in the AUC in the ability to diagnose early lung cancer when combining the AI risk score with radiological characteristics. However, when using only this information, the sensitivity of the first two models was over 80% in the two cohorts, but the specificity rates were only between 62.52% and 65.96%. As indicated by the AUCs, model 3, which included clinical characteristics, radiological characteristics, and the liquid biopsy result, performed better than models 1 and 2, which only considered information provided by LDCT with and without the assistance of AI. The highest diagnostic value was attained in a model that combined clinical and radiological characteristics, AI analysis of LDCT data, and liquid biopsy results with over 80% sensitivity and specificity. Compared to models 1 and 2, the enhancement in specificity in models 3 and 4, which combined multiple predictors, namely, liquid biopsy data and clinical data, has the potential to reduce harmful side effects such as pneumothorax and bleeding, which may be caused by invasive biopsy, suggesting that the liquid biopsy result and LDCT may complement one another. These findings provide evidence that using a classifier with a broad range of validated predictors may improve the diagnostic accuracy for early lung cancer.

The use of AI in cancer diagnosis is gaining acceptance and has been investigated for its ability to assist physicians in early lung cancer detection. AI can assist clinicians in expediting the interpretation of different pathological diagnoses and reducing the mental fatigue caused by classifying a large number of medical images (26). With the increasing incidence of lung cancer in rural China and the lack of skilled physicians (27), AI may be an excellent tool for clinicians to use as a supplement to the interpretation of LDCT images. To date, the performance metrics of AI in diagnosing lung cancer have not been verified in either retrospective data, such as the NLST dataset (28–30), or relatively small datasets (31). This prospective study evaluated the diagnostic power of AI in a large cohort of 728 patients with validated lung cancer histopathology.

We chose the 4-color FISH assay for this study as we had previously demonstrated that this assay was superior to serum protein biomarkers such as carcinoembryonic antigen, neuron-specific enolase, and cytokeratin 19 fragment (32). Furthermore, certain assays for circulating tumor cells, circulating tumor DNA, and exosomes have been measured in research studies (33, 34); however, most of these assay technologies are insensitive to early-stage lung cancer and are not commercially available for detecting early lung cancer (35–37). The FISH-based liquid biopsy assay was approved for commercial use by the China National Medical Products Administration. The performance of the test was verified in a 10-year study conducted in the USA with an accuracy rate of 94.2% in 207 participants (107 patients with lung cancer, 26 patients with benign nodules, and 80 control participants) who were at high risk of developing lung cancer (25). Additionally, in a study conducted in China, the same assay yielded sensitivities of 66.7 and 73.0% for 339 participants with pure ground-glass nodules and mixed ground-glass nodules who were diagnosed with early NSCLC (32). The results of these studies indicate that the FISH assay is a reliable tool for early lung cancer diagnosis.

According to the American College of Chest Physicians guidelines, upper lobe location is a risk factor for lung cancer, as indicated by the Mayo Clinical Model, with an odds ratio (OR) of 2.2 (38). The OR of upper lobe location in our study was 1.750 (p = 0.005). This finding may indicate that, in the Chinese population, the presence of pulmonary nodules located in the upper lobe is associated with a higher risk of malignancy than those discovered in other lobes, even when considering the high prevalence of pulmonary nodules in the upper lobe secondary to tuberculosis. In addition, the AUC of our best performance model was 0.895 in the independent validation cohort, which was superior to that of the Mayo Clinic Model (0.772) and the VA model (0.740). These results demonstrate that it is necessary to develop an early lung cancer classifier based on data retrieved from a Chinese population.

Our study has some limitations. First, because the participants traveled from various locations in the country prior to visiting our outpatient clinics to seek help in evaluating their nodule status, we were unable to calculate the disease prevalence in the general population. Patients in China are more likely to visit tertiary hospitals in big cities after they have discovered pulmonary nodules by LDCT in their hometowns. Since electronic health records are not shared between hospitals, we cannot track back how many people went for lung cancer screening before those with an intermediate and high risk of lung cancer went to the 12 outpatient clinics in the main cities of China. Second, our study cohort was small compared to national-scale data sets, such as those derived from the NLST and the Dutch–Belgian Randomized Lung Cancer Screening Trial (NELSON), and therefore might not be representative of the early lung cancer characteristics of the entire Chinese population; however, this is a diagnostic study and not a screening study in the general population, we have included individuals with positive LDCT results and evaluated as intermediate and high-risk for lung cancer by physicians in the usual care routine.

In the future, we hope to apply this methodology in a prospective study with a larger sample size to continue to validate and refine our classifier to improve early lung cancer diagnosis. Given the high number of pulmonary nodules discovered by LDCT scans, many patients with nodules might need to wait for a long period for physicians to interpret CT images to evaluate the significance of these lung nodules. If nodules are suspicious for malignancy, these patients may require surgical excision, biopsy, or stereotaxic radiation; however, if benign, these patients should undergo serial CT scans. The use of a multivariate lung cancer prediction model as proposed herein can help relieve the patients’ anxiety by reducing the follow-up time to a definitive diagnosis if the risk score is high or delaying the follow-up time to less frequent LDCT scans if the classifier returns a low-risk score. This will help to streamline clinical decision making by physicians for a large number of patients. We believe that a noninvasive tool such as this classifier will be a good complementary tool for physicians in the assessment of early lung cancer.

## Data Availability Statement

The original contributions presented in the study are included in the article/supplementary material. Further inquiries can be directed to the corresponding authors.

## Ethics Statement

The studies involving human participants were reviewed and approved by the Ethics Committee of Zhongshan Hospital, Fudan University. The patients/participants provided their written informed consent to participate in this study.

## Author Contributions

Conception and design: JS and CB Development of methodology: MY, LT, XZhe, HW, HZ, XZhu, CZ, PZ, YaW, QW, LB, ZC FK, YuW, MF, and XY. Provision of study materials: DY, ZL, QZ, ZW, SH, LS, NZ, ZY, JZ, XZha, and JS. Statistical analysis: YL, Revised the manuscript: RK, JS, and CB. All authors listed have made a substantial, direct, and intellectual contribution to the work and approved it for publication.

## Funding

This study was supported by the Program for the Guangdong Introducing Innovative and Entrepreneurial Teams (2019ZT08Y297) and the Shanghai Engineering & Technology Research Center of the Internet of Things for Respiratory Medicine (20DZ2254400).

## Conflict of Interest

Authors XY and JZ are employees of Zhuhai Sanmed Biotech Ltd. RK is a consultant of Sanmed Biotech Ltd.

The remaining authors declare that the research was conducted in the absence of any commercial or financial relationships that could be construed as a potential conflict of interest.

## Publisher’s Note

All claims expressed in this article are solely those of the authors and do not necessarily represent those of their affiliated organizations, or those of the publisher, the editors and the reviewers. Any product that may be evaluated in this article, or claim that may be made by its manufacturer, is not guaranteed or endorsed by the publisher.
